# Chikungunya: vaccines and therapeutics

**DOI:** 10.12688/f1000research.12461.1

**Published:** 2017-12-08

**Authors:** Kothila Tharmarajah, Suresh Mahalingam, Ali Zaid

**Affiliations:** 1 Institute for Glycomics, Griffith University Gold Coast, Southport, Queensland, Australia

**Keywords:** chikiungunya, vector control, vaccine development

## Abstract

Chikungunya virus (CHIKV) has come to prominence as a global, re-emerging pathogen over the last two decades, progressing from sporadic, remote outbreaks to worldwide explosive epidemics. From contained, though considerable, outbreaks in the southern Indian Ocean, parts of South America and the Caribbean, CHIKV continues to be a significant pathogen in Southeast Asia and India. CHIKV circulates during epidemics through an urban mosquito-to-human transmission cycle, and with no available treatments or licensed vaccines to specifically target CHIKV disease, limiting transmission relies on vector control, which poses significant challenges, especially in developing countries. This review summarizes the current findings and progress in the development of safe, effective and affordable therapeutics and vaccines for CHIKV disease.

## Background

Chikungunya virus (CHIKV) is an arthritogenic alphavirus of the *Togaviridae* family transmitted to humans by female *Aedes* mosquito vectors. Chikungunya viral disease (CHIKVD) affects all age groups, and symptoms present as fever, rash and severe, debilitating polyarthralgia that often progresses to a chronic stage ^1, 2^. A significant manifestation is the loss of function of tarsal joints (hands, fingers and elbows), rendering simple activities extremely painful. In rare cases, CHIKVD can be fatal, particularly in the elderly, neonates and individuals with pre-existing conditions such as diabetes and cardiovascular disorders ^3– 5^.

Unlike other alphaviruses, CHIKV sustains urban transmission between humans and mosquitoes causing large, sporadic epidemics such as those seen during the 2004–2006 outbreaks in Lamu Island ^6^, La Reunion ^7^ and Southern India ^8^. From localised outbreaks in Southern and Southeast Asia, the epidemic has disseminated—potentially enhanced by international travel—and caused small outbreaks in Europe ^9, 10^ and North America ^11, 12^ while severely affecting territories in South and Central America and the Caribbean ^13, 14^, and more than a million cases are reported annually. With elevating global temperatures facilitating the spread of *Aedes* mosquito, the strong potential for other mosquito species to carry CHIKV, poor vector controls and a lack of licensed vaccines or therapeutics, the risk for future epidemics stretching beyond the geographical confines of tropical, developing areas is increasing significantly. In this article, we explore recent findings and progress in the development of therapeutics and vaccines against CHIKV.

## Current treatments

Current therapies for CHIKV-infected patients with arthritis/arthralgia mainly involve management of pain and inflammation using non-steroid anti-inflammatory drugs (NSAIDs), along with fluid intake to prevent dehydration. NSAIDs remain the primary approach for disease management as the use of aspirin may pose a risk of bleeding and potentially developing Reye’s syndrome, and the administration of corticosteroids is likely to cause immunosuppression and exacerbate the disease. In patients who exhibit limited response to NSAIDs or those with chronic CHIKVD, disease-modifying anti-rheumatic drugs (DMARDs) such as methotrexate, hydroxychloroquine and sulfasalazine have been reported to alleviate pain and joint swelling ^15, 16^. As there are no licensed antivirals or vaccines available for CHIKVD, there is an imperative need for the development of novel and potent drugs and vaccines.

## Therapeutics

### Antivirals

Antivirals act by targeting specific stages in the virus replication cycle, thereby inhibiting viral entry, replication and budding. A majority of anti-CHIKV molecules reported have been identified by testing compounds with already established antiviral properties. Favipiravir (T-705), an antiviral agent approved in Japan for treatment against influenza virus, together with its de-fluorinated analogue T-1105, inhibited CHIKV replication *in vitro* ^17^. Furthermore, CHIKV-infected AG129 mice treated orally with T-705 displayed less severe neurological disease and more than 50% reduction in mortality rate ^17^. Another broad-spectrum antiviral drug, ribavirin, traditionally used to treat respiratory syncytial virus in infants ^18^ and chronic hepatitis C virus in combination with interferon-alpha (IFN-α) ^19^, exhibited inhibitory effects on *in vitro* CHIKV replication ^20^. Though somewhat effective on its own, a potent inhibitory effect on CHIKV replication was observed when ribavirin was used in combination therapy with IFN-α ^20^. Similar broad-spectrum drugs that have shown *in vitro* antiviral activity against CHIKV include Arbidol ^21^, licensed in Russia and China for the treatment of influenza virus-infected patients ^22^, and suramin, licensed for treatment against trypanosomiasis ^23^. However, the effect of these antivirals—whether it be prophylactic or therapeutic—is yet to be characterised using *in vivo* models of CHIKV infection.

In addition, harringtonine, a plant alkaloid compound, and its methylated stable analogue homoharringtonine, used in the treatment of chronic myeloid leukaemia, inhibited CHIKV replication *in vitro* ^24^. Treatment with the compound reduced viral RNA production and the synthesis of viral non-structural protein nsP3 and structural E2 protein. Similarly, mycophenolic acid, used clinically as an immunosuppressant in organ transplantations, has been shown to impair CHIKV replication *in vitro*. A similar inhibitory effect was seen with 6-azauridine, an antimetabolite, on *in vitro* CHIKV replication ^25, 26^.

More recently, several novel small-molecule antiviral compounds that interfere with CHIKV replication *in vitro* have been identified. Compounds that selectively target nsP1 and nsP2, which possess enzymatic properties essential for viral replication, have been found to inhibit viral replication ^27– 29^. Similarly, nucleoside analogue β-D-N ^4^-hydroxycytidine (NHC), previously shown to inhibit hepatitis C virus replication, has been shown to selectively inhibit CHIKV replication *in vitro* and was found to be more potent than favipiravir and ribavirin ^30^.

Although a number of antiviral compounds have been identified to be effective against CHIKV *in vitro* and in specific animal models, further research is needed to determine the effectiveness and safety of these molecules against CHIKV replication and CHIKV-induced disease *in vivo* before considering their use in a clinical setting.

Several of the abovementioned broad-spectrum antivirals have passed clinical trials in humans and are currently in use as therapeutics for other conditions. Should further studies confirm reliable, effective anti-CHIKV activity *in vivo*, these compounds could prove to be the most practical way forward as a short-term, emergency intervention strategy: the market readiness and established safety profile of some of these compounds would make them promptly accessible in the event of a widespread CHIKV epidemic.

### Antibodies

Prophylactic and therapeutic treatments using neutralizing monoclonal antibodies (mAbs) in CHIKV animal models have dominated the experimental field in the past 4 years, and several studies have shown mAbs to be highly effective in animal models of CHIKV infection. Human neutralizing mAbs directed against the E1 and E2 domains were shown to substantially reduce lethality in CHIKV-infected AG129 and RAG2 ^−/−^ mice, which are highly susceptible to CHIKV infection ^31^. In a related study, neutralizing mAbs specifically targeting the E2 domain reduced viral load and foot swelling in infected adult wild-type mice in a prophylactic setting and in a therapeutic setting when administered 8 or 18 hours post-infection to neonatal wild-type mice ^32^. In a parallel study, wild-type adult AG129, RAG2 ^−/−^ and IFNAR1 ^−/−^ mice—all susceptible to CHIKV infection—treated with neutralizing antibodies before infection with CHIKV displayed similar levels of protection from lethal disease ^33– 35^. Additionally, prophylactic treatment of RAG1 ^−/−^ mice—which lack B and T cells and thus exhibit persistent CHIKV infection—with anti-CHIKV mAbs resulted in lower viral titers in muscle tissue and sera ^35^.

Beyond the use of murine models of disease, treatment of infected rhesus macaques with SVIR001, an engineered mAb that mimics neutralizing anti-CHIKV human mAb 4N12, led to rapid viral clearance and reduced severity of joint inflammation in comparison with an isotype control antibody ^36^. SVIR001 is believed to modulate the inflammatory pathway by downregulating the activation of immune cells and expression of pro-inflammatory mediators while maintaining adaptive immune responses against CHIKV. Moreover, it stimulated effective clearance of the virus at the site of infection, resulting in reduced viral load. In a separate study, a combination therapy with CTLA4-Ig (abatacept)—a biological DMARD that blocks T-cell co-stimulation—and the 4N12 mAb abolished periarticular swelling and significantly reduced pro-inflammatory chemokines and cytokines, which in turn reduced leukocyte infiltration into tissues ^37^. This combination therapy effectively reduced joint inflammation even when administered several days after infection and could be of great benefit in situations where CHIKV-infected patients present several days after the onset of symptoms, as is often the case.

Taken together, these studies suggest that neutralizing mAbs can be effective therapeutically and prophylactically. In particular, the prophylactic approach could be recommended for individuals at increased risk of CHIKV infection, such as pregnant women, in light of reports of mother-to-child transmission ^38^, or patients with underlying conditions known to intensify the disease, such as diabetes mellitus, cardiac failure and chronic obstructive pulmonary disease ^39^. However, prophylactic approaches would be limited as a short-term prevention strategy for patients living in CHIKV-endemic regions, as regular boosters may be required for continued protection, which would prove to be a financial burden. Although antibody-based therapies are favoured because of their high specificity, significant shortcomings remain: hypersensitivity, short serum half-life requiring multiple administrations, limitation in target tissue accessibility and high production costs. Significant research and development are being focused on neutralizing antibody-based approaches to generate a cost-effective and broadly accessible antibody that provides long-lasting protection.

## Vaccines

Like all vaccines under development, CHIKV vaccine candidates developed via various technologies - such as live-attenuated virus vaccines, inactivated viral vaccine, recombinant viral vaccines, chimeric-alphavirus candidates, DNA vaccines and virus-like particles (VLPs) - require an optimal balance between immunogenicity and safety. Of the various vaccine candidates that are in pre-clinical studies, two (MV-CHIK and VRC-CHKVLP059-00-VP) have successfully completed phase I clinical trials ^40– 42^.

The MV-CHIK vaccine is a recombinant measles virus (MV) that expresses CHIKV surface proteins from the La Reunion ECSA CHIKV strain ^40^. In pre-clinical studies, this vaccine protected mice from lethal CHIKV challenge after one or two immunizations ^40^. In a phase I trial (Vienna) ^41^, 42 healthy adults (18–45 years) were evaluated with three different immunization doses. MV-CHIK elicited a strong neutralizing antibody response in all subjects following the first dose, which was further boosted after a second immunization with no reports of adverse events. Importantly, MV-CHIK showed no significant immunogenic response to pre-existing MV exposure. Phase II trials, where dosage levels are compared by assessing immunogenicity, safety and tolerability, began in August 2016 ^43^ and are expected to be completed in June 2018.

In addition, a VLP vaccine, VRC-CHKVLP059-00-VP, the first VLP candidate to reach phase I trials, induced a strong immune response and protected against CHIKV infection in mice and non-human primates ^44– 46^. The vaccine is composed of VLPs expressed on host cell–derived membrane transfected with a plasmid encoding CHIKV structural E1, E2 and capsid (C) proteins. The phase I study tested 25 healthy adults at three intramuscular immunization doses (10, 20 and 30 µg) administered at weeks 0, 4 and 24 ^47^. The vaccine was well tolerated and produced high antibody titers against the eastern, central and southern African OPY1 CHIKV outbreak strains. Neutralizing antibody titers achieved after the third dose were reported to be comparable to titers found in recovering CHIKV-infected patient sera. A multi-centre phase II trial commenced in September 2015 ^48^ with an estimated completion date in December 2017.

The VLP-based vaccines lack a live and replicating virus; instead, they consist of structural proteins of the virus which, alone or in combination, can elicit a protective immune response. It must be noted that a potential shortcoming of the VLP-based vaccines is that multiple immunizations or the use of adjuvants may be required to provide sufficient long-term immunity; this could enhance reactogenicity, impair the vaccine’s tolerability and increase the manufacturing cost of the vaccine.

Recently, vaccines based on mutation-attenuated virus have been the focus of pre-clinical studies. A study by Taylor *et al*. revealed that site-directed mutations in the nuclear localisation sequence (NoLS) of the N-terminal region of CHIKV capsid protein impaired viral replication *in vitro* and protected adult mice 30 days post-immunization from wild-type CHIKV infection ^49^. This attenuated CHIKV-NoLS vaccine also proved to be cross-protective by reducing peak viremia in immunized mice challenged with a related arthritogenic alphavirus, Ross River virus. Although traditional attenuated live viral vaccines are highly efficient, new approaches using chimeric virus vaccines are proving to be useful. A chimeric viral vaccine combining Eilat virus—an insect-specific alphavirus—and CHIKV structural proteins induced a rapid, long-lasting neutralizing antibody response in C57BL/6 and immunocompromised IFNα/βR ^−/−^ mice after a single dose ^50^. This single-dose efficacy of the vaccine also stimulated similar immunogenicity and protected against CHIKV infection in cynomolgus macaques.

## Conclusions

CHIKV outbreaks are sporadic and episodic, and the dissemination of CHIKV to the Caribbean and South and Central America is a strong reminder of the importance and urgency of effective treatments and vaccines. Vaccines are the most cost-effective method to prevent disease. While VLP- and MV-based vaccines have higher safety profiles, the cost of production and the potential requirement for additional boosters may discourage widespread use in resource-poor countries where CHIKV is endemic, such as India, Bangladesh and southern Indian Ocean islands. Various promising antiviral inhibitory drugs have been reported to be effective against CHIKV replication *in vitro*, and some studies have shown efficacy using *in vivo* alphavirus infection models. Similarly, the administration of neutralizing antibodies may prove to be highly efficient, although high manufacturing costs and the potential risks of hypersensitivity, immunosuppression and infections constitute significant shortcomings. The effectiveness of therapeutics and vaccine candidates from current research is dependent on capital investment, product stability and the long-term public health measures and preventative strategies in endemic regions. Moreover, substantial gaps in our understanding of the underlying mechanism of viral replication and acute/chronic disease clearly indicate that more research is needed to develop effective therapeutic and preventative strategies.
